# Cognitive functioning in a group of adolescents at risk for psychosis

**DOI:** 10.3389/fpsyt.2022.1075222

**Published:** 2022-12-01

**Authors:** Caroline Ranem Mohn-Haugen, Christine Mohn, Frank Larøi, Charlotte M. Teigset, Merete Glenne Øie, Bjørn Rishovd Rund

**Affiliations:** ^1^Research Department, Vestre Viken Hospital Trust, Drammen, Norway; ^2^Department of Psychology, University of Oslo, Oslo, Norway; ^3^NORMENT Centre, Institute of Clinical Medicine, University of Oslo, Oslo, Norway; ^4^Psychology and Neuroscience of Cognition Research Unit, University of Liège, Liège, Belgium

**Keywords:** at risk for psychosis, adolescence, cognition, schizophrenia, birth cohort

## Abstract

Cognitive deficits are a core feature of schizophrenia, and impairments are present in groups at-risk for psychosis. Most at-risk studies include young adults and not younger age-groups, such as adolescents. Participants are usually help-seeking individuals, even though risk factors may also be present in non-help seeking adolescents. We aim to explore cognitive functions in a group of non-help-seeking 15-year-old adolescents at risk for psychosis compared to age- and gender matched controls, including particular focus on specific cognitive domains. Hundred participants (mean age = 15.3) were invited after completing the 14-year-old survey distributed by the Norwegian Mother-, Father- and Child Study. At-risk adolescents were selected based on high scores on 19 items assessing both psychotic experiences and anomalous self-experiences. Matched controls were selected from the same sample. Cognitive functioning was assessed using the MATRICS Consensus Cognitive Battery and IQ using Wechsler’s Abbreviated Test of Intelligence. We found that the adolescents at-risk for psychosis had significantly poorer scores than controls on the composite score of the MCCB. IQ scores were also significantly lower in the at-risk group. The results highlight general cognitive deficits as central in a group of non-help-seeking adolescents at-risk for psychosis. Results indicate that the development of cognitive impairments starts early in life in at-risk groups. It is still unclear whether specific cognitive domains, such as verbal learning, are related to psychotic symptoms or may be specifically vulnerable to symptoms of depression and anxiety.

## Introduction

Cognitive deficits are a core feature of schizophrenia spectrum disorders (SSD), and impairments are also present in at-risk groups before onset of illness. In general, the evidence suggests that about two thirds of individuals with SSD experience significant cognitive impairments (1, 2). There has been much debate surrounding the nature and development of cognitive impairments in at-risk groups and its relationship with risk for psychosis, transition to SSD and functional outcomes [e.g., (3, 4)]. Both neurodevelopmental and neurodegenerative theories have been posited as probable models explaining the development of SSD (5, 6). The neurodevelopmental model explains how subtle impairments in cognition start early in life and develop in a fashion that indicates abnormal maturation of the nervous system that is a risk factor for developing SSD, where cognitive deficits stabilize after onset of illness (7, 8). The neurodegenerative model explains cognitive deterioration as a result of progressive psychotic illness, where cognitive decline continues after illness onset (9). The evidence for decline after first-episode psychosis is mixed (10). When looking at a wide variety of studies where individuals have been cognitively tested before onset of SSD, evidence indicates that specific cognitive deficits appear at different stages in life before illness onset. For instance, verbal impairments have been detected as early as 3 years of age and non-verbal and executive impairments later in adolescence (11). Knowledge of when specific impairments are identifiable is important for understanding the pathogenesis of SSD pre-onset.

In research on at-risk individuals, clinical high-risk (CHR) criteria are widely used. CHR is comprised of individuals who present with either impaired functioning *and* family history of SSD, transient psychotic symptoms, or subthreshold psychotic symptoms (12). The latest and largest meta-analysis of cognition in CHR as compared to healthy controls found that the CHR group performed worse than healthy controls across all cognitive domains (13). They also found that longitudinal transition to psychosis was associated with deficits in verbal learning, visual memory, processing speed and attention or vigilance, as well as general intelligence (IQ). The mean age of participants across the 78 independent studies included in the meta-analysis (13) was 20.2 years, and in a previous review it was 18.5 years (11). These encompassing studies highlight the paucity of studies investigating cognition in adolescents at risk for psychosis. Furthermore, CHR research typically involves individuals seeking help for their mental health problems. However, identifiable risk factors in older and help-seeking individuals may be different than for younger, non-help seeking adolescents.

A second group at increased risk for developing SSD is familial-high risk (FHR), meaning individuals with close relatives diagnosed with SSD. Recently, a study was published, and results showed that FHR children between ages 7 and 11 demonstrate widespread cognitive impairments compared to typically developing controls (14). FHR research is based on well-known risk-factors that are associated with impaired cognitive function and could be considered a separate at-risk population. Population cohort approaches, although valuable, are few and only identify at-risk individuals in retrospect (15–17). Moreover, it would be valuable if studies employed consensus batteries. Using consensus batteries makes for easier comparisons across studies, and ensures that a broad selection of cognitive domains are tested.

To our knowledge, only one previous study included both children and adolescents ages 9–16 at either FHR for SSD or at elevated clinical risk due to psychotic-like symptoms, emotional difficulties, and developmental delays (18). The participants were not included based on help-seeing behavior. Results identified deficits in verbal working memory, inhibition/switching executive functions, vocabulary, word reading, numerical operations and category fluency in the at-risk group when compared to typically developing controls. Furthermore, the authors identified developmental lag in spelling and developmental delay in category fluency, and in visual and verbal memory. However, the study had a small sample size, particularly at the third assessment at 14–16 years (*N* = 25). Taken together, the existing evidence suggests early- and mid-adolescence as important time points where cognitive impairments may be identifiable in at-risk groups, and thus targets for remediation efforts. However, studies focusing on young adolescents are almost non-existing and existing results require replication.

In addition to the challenges outlined above, the clinical picture of at-risk populations is often complicated by comorbid symptoms of anxiety and depression (12). Depression and anxiety have been shown to have a negative impact on cognitive function (19, 20). In at-risk groups it has been found that depression and anxiety may be unrelated to risk of transition to SSD (21, 22), but may be associated with poorer outcomes of global functioning (23). Despite the knowledge that comorbidity is common in this population, its associations with cognitive function have been understudied. One study found that baseline major depressive disorder (MDD) in a CHR population is independently related to poorer verbal memory and higher verbal fluency (24).

Also, adolescence is a period marked by a brain in rapid development, as well as behavioral and cognitive systems that mature at different stages and at different paces (25). In particular, the maturation of the prefrontal cortex is central in adolescence. Its development is protracted throughout adolescence and disturbances in prefrontal structures are strongly related to later SSD (26). This underlines the importance of detecting cognitive deficits as early as possible in at-risk groups. This will allow for more informed timing for interventions targeting cognitive deficits, which may be more beneficial in the at-risk state compared to more advanced illness stages (27). Ultimately, this research can contribute to the understanding of how cognitive impairments in adolescence relates to a risk of transition to SSD, psychiatric comorbidity, and functional outcomes.

The current study included young adolescents at-risk for psychosis compared to age- and gender matched, randomly selected controls, and investigated specific cognitive domains using the Matrics Consensus Cognitive Battery (MCCB) (28). This study enriches the existing literature by targeting youth in a large population-based cohort, the Norwegian Mother-, Father- and Child Study (MoBa) (29). In this article we aim to explore cognitive functions in a group of non-help-seeking 15-year-old adolescents at risk for psychosis compared to age- and gender matched controls, including particular focus on specific cognitive domains.

## Materials and methods

The present study is a sub-study of the Norwegian, Father and Child Cohort Study.

### The Norwegian Mother, Father and Child Cohort Study

The Norwegian Mother, Father and Child Cohort Study (MoBa) is a population-based pregnancy cohort study conducted by the Norwegian Institute of Public Health. Participants were recruited from all over Norway from 1999 to 2008. The cohort includes approximately 114,500 children, 95,200 mothers and 75,200 fathers, and 41% of all eligible pregnant women agreed to participate. The current study is based on MoBa data files released for research in March of 2022. The establishment of MoBa and initial data collection was based on a license from the Norwegian Data Protection Agency and approval from The Regional Committees for Medical and Health Research Ethics. The MoBa cohort is currently regulated by the Norwegian Health Registry Act. The present study was approved by The Regional Committees for Medical and Health Research Ethics (ref. 2017/342). Permission to access the 14-year-old survey as part of the MoBa project was granted by the Norwegian Data Inspectorate (ref. 16/01296-2/GRA). The study is being conducted with basis in Vestre Viken Hospital Trust in Norway.

### Selection of individuals at-risk for psychosis

At age 14, the adolescents are invited to participate in a survey consisting of about 200 items asking about a wide variety of mental health and well-being issues, including 19 items concerning psychotic experiences and anomalous self-experiences (ASE). The 19 items were selected from two instruments that have been used to identify adolescents at-risk for psychosis and to predict later transition to SSD (30, 31). The items were included based on agreements between MoBa and leading experts in the field of psychosis research. Items that are the most sensitive at identifying adolescents at-risk for psychosis prospectively, in a population cohort, were utilized.

Sixteen of the 19 items assess positive psychotic symptoms and stem from the Community Assessment of Psychic Experiences questionnaire (CAPE-42) (32). If participants confirm having experienced an item (i.e., reply “sometimes,” “often,” or “nearly always”), they are asked to assess the degree of distress associated with this experience ranging from “Not distressed,” “A bit distressed,” “Quite distressed,” and “Very distressed.” The CAPE was designed to identify psychotic experiences in the general population. It has been found to be useful as a screening tool to detect individuals who fulfill criteria for psychotic disorders (33). Furthermore, the 15-item version of CAPE, the CAPE-15 (all these 15 items are included in our 16-item version), has been found to be suitable for accurately classifying and identifying psychotic experiences in adolescents in a general population (34). In addition, the CAPE-15 has been found to be able to detect youth at ultra-high risk (UHR), as identified with the Comprehensive Assessment of At-Risk Mental States (CAARMS) (31). Using a cut-off score of 1.47 for both scores, CAPE-15 showed a sensitivity of 77% for frequency scores and 73% for distress scores and specificity of 58% for frequency scores and 63% for distress scores, meaning that only individuals who experience frequent symptoms and are distressed by them would be selected as ultra-high risk. Using a cut-off of 4‰ highest scores, we aimed to ensure the same.

Three of the 19 items assess ASE and are based on the Examination of Anomalous Self Experience (EASE) (35), a comprehensive clinical interview. The three items included in this study were formulated by experts on EASE and ASE in SSD (36). Each item contains two parts: first asking the participant if they have had a particular self-disturbance experience, followed by asking them if it was very distressful and had a very negative impact on the person. Alternatives were “Not the case,” “Sometimes the case,” and “Fits very well.” The inclusion of items asking about ASE were based on the fact that these experiences are considered to be core features of SSD, both during pre-onset phases and in established illness (37–39). Furthermore, ASE, as measured by the EASE clinical interview, have been found to predict transition from an at-risk state to established illness (30).

**FIGURE 1:**
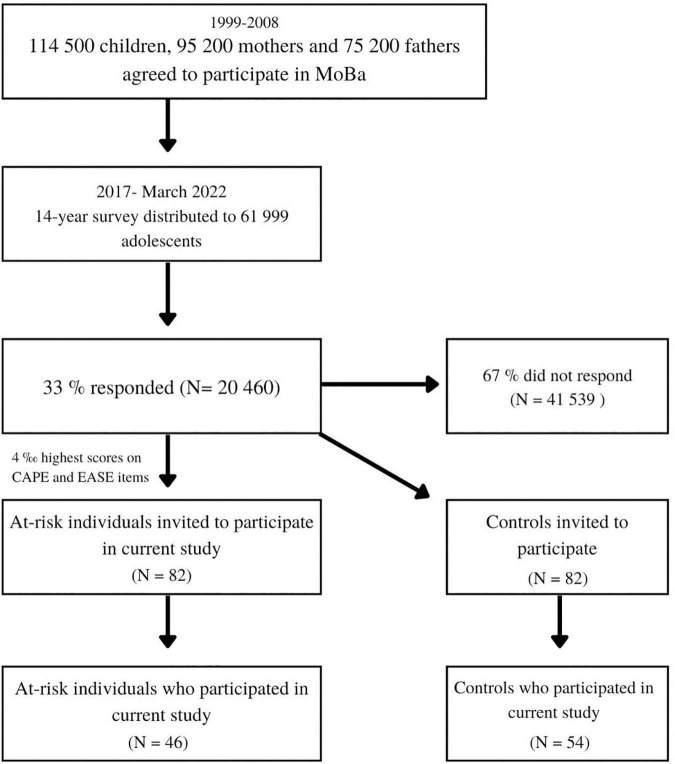
Flowchart of inclusion.

An outline of the inclusion process is presented in Figure 1. As of March 2022, the survey has been distributed to 61,999 adolescents, of which 33% have responded. The at-risk group was defined as the 4‰ who had the highest scores on the 19 items. The cut-off of 4‰ was chosen in cooperation with the Regional Committee for Medical and Health Research (REK) to increase the probability of selecting youth at-risk for psychosis, i.e., to reduce the rate of false positives. A letter of invitation was sent by mail to both the parents and adolescent and included general information about the aims of the project. Written consent was provided by parents and informed consent was provided from the adolescent at the time of testing. Adolescents who agreed to participate in the current study were interviewed and tested on average between 6 and 8 months after completing the survey, with some delays during the COVID-19 pandemic. Due to the utilization of psychosis-specific items, we define this group as at-risk for psychosis, but not as clinical- or ultra-high risk as defined by clinical interviews such as CAARMS. As this is an on-going and prospective study, it is not known how many of the at-risk adolescents’ transition to SSD, or other mental disorders. The control group consisted of randomly selected gender- and age-matched controls from the same sample. The first 100 participants who were included consisted of an at-risk group (*N* = 46) and a gender- and age- matched control group (*N* = 54) (mean age = 15.4).

### Clinical assessment and cognitive testing

The Mini International Neuropsychiatric Interview (MINI) (40) was used to assess symptoms of psychopathology. Cognitive functioning was assessed with the MCCB (28) and general intelligence with 4 subtests of the Wechsler Abbreviated Scale of Intelligence (WASI) (41). The MCCB covers core cognitive functions, including processing speed, attention/vigilance, working memory, verbal learning, visual learning, reasoning and problem solving, and social cognition, whilst the WASI estimates general intelligence.

### Procedure

Participants were assessed by two clinical psychologists who were trained in standardized neuropsychological testing. Each session was conducted either in the participants’ home or a suitable equivalent and lasted between 2 and 3 h, including breaks. Participants received monetary compensation. The assessors were not informed of the participants’ risk status beforehand.

### Statistical analyses

All statistical analyses were conducted using The Statistical Package for the Social Sciences (IBM SPSS Statistics for Windows, version 28) (42). All tests were 2-tailed, and the methods used were *t*-tests and ANOVAs for group comparisons with continuous data and chi-square-tests for group comparisons of categorical data. Due to multiple comparisons in a relatively small sample, all *p*-values were corrected using the Benjamini–Hochberg procedure. The level of significance was set at *p* = 0.05. Partial eta squared provided the effect size for the group differences. There was a skewness of −1.07 for visual learning in the control group and −1.35 for visual learning and −1.57 for reasoning in the at risk group, none of which have implications for the results. As the available MCCB norms were based on American adults (20–24 years), we calculated T-scores based on the raw scores of our sample with a mean of 50 and an SD of 10. WASI scores are based on Norwegian norms (41).

## Results

Demographic characteristics of at-risk participants and controls are presented in Table 1. The results show that the two groups did not differ in terms of sex distribution, age or years completed education. Table 1 also presents standardized scores for the two groups on each cognitive domain. Differences between the two groups were statistically significant for the composite score of the MCCB, Verbal IQ and Fullscale IQ (FSIQ).

**TABLE 1:** Demographics and T-scores for WASI and MCCB batteries.

	At-risk (*N* = 46)		Controls (*N* = 54)		Test statistics		
**Demographics^a^**							
Sex (female)	42 (91.0%)		49 (91.0%)		χ^2^ = 0.01		ns
Age (year)	15.4 (0.5)		15.3 (0.5)		t = 0.89		ns
Years education	9.8 (0.5)		9.6 (0.7)		t = 1.10		ns

**T-scores**	**Mean**	**SD**	**Mean**	**SD**	***F***	*p* *	**η^2^**

**WASI**							
FSIQ^b^	99.5	10.1	105.9	11.7	8.30	**0.036**	0.08
Verbal IQ	93.8	12.1	101.8	13.0	10.10	**0.018**	0.09
Performance IQ	104.9	12.5	109.0	11.1	2.89	0.109	0.03
**MCCB**							
Processing speed	48.6	7.3	51.2	7.6	3.00	0.090	0.03
Attention	48.3	10.7	51.4	9.3	2.30	0.127	0.02
Working memory	49.4	9.3	50.5	7.2	0.44	0.181	0.00
Verbal learning	47.7	11.7	51.9	7.9	4.58	0.072	0.05
Visual learning	48.4	11.2	51.3	8.7	2.14	0.145	0.02
Reasoning	49.5	10.9	50.4	9.2	0.19	0.200	0.00
Social cognition	48.6	9.7	51.2	10.2	1.69	0.163	0.02
Composite score	48.4	5.6	51.1	5.0	6.62	**0.054**	0.06

^a^Age and education in mean (SD). ^b^FSIQ: estimated full scale IQ. *All *p*-values have been Benjamini–Hochberg corrected.

Bold values represent the significant values.

**FIGURE 2:**
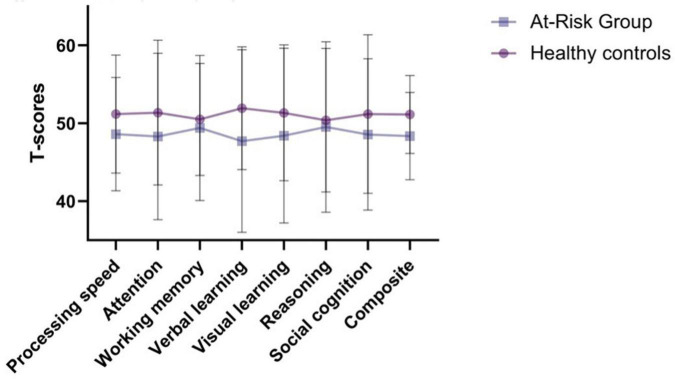
MCCB (mean, SD).

The MCCB results are presented in Figure 2. The figure illustrates a general pattern where the at-risk group performed below controls, although not significantly so, on most domains. It also highlights the substantial dispersion in scores for each domain in both groups.

When examining the raw scores of the specific tests, there were significant differences on the HVLT-R, and the Vocabulary and Similarities subtests of the WASI (see Table 2).

**TABLE 2:** Raw scores, neuropsychological tests.

	At-risk (*N* = 46)		Healthy controls (*N* = 54)		*F*	*p* *	η^2^
	Mean	SD	Mean	SD			
Vocabulary	46.7	6.8	50.3	6.5	7.29	**0.028**	0.07
Similarities	31.1	5.3	34.1	5.5	7.52	**0.014**	0.07
Block design	51.0	13.4	53.0	11.5	0.65	0.157	0.00
Matrix reasoning	27.6	3.7	29.0	3.5	4.00	0.057	0.04
TMT-A	24.9	7.8	22.2	6.5	3.59	0.071	0.04
Symbol Coding	55.4	9.1	56.4	10	0.28	0.171	0.00
Category fluency	24.6	5.4	26.3	6.2	2.15	0.093	0.02
CPT-IP	1.9	0.6	2.0	0.6	2.30	0.085	0.02
LNS	13.7	3.3	14.3	2.8	0.92	0.142	0.00
Spatial span	18.0	3.1	18.0	2.8	0.02	0.200	0.00
HVLT-R	26.4	4.8	28.1	3.2	4.48	**0.042**	0.05
BVMT-R	29.0	5.8	30.5	4.5	2.14	0.114	0.02
Mazes	21.4	4.4	21.7	3.7	3.03	0.185	0.00
MSCEIT	88.8	8.5	91.1	8.9	1.69	0.128	0.02

MCCB, MATRICS consensus cognitive battery; TMT-A, trail making test A; CPT-IP, continuous performance test—identical pairs; LNS, letter number sequencing; HVLT-R, Hopkins verbal learning test—revised; BVMT-R, brief visual memory test—revised; MSCEIT, mayer-salovey-caruso emotional intelligence test. *All *p*-values have been Benjamini-Hochberg corrected.

Bold values represent the significant values.

Based on the MINI, the at-risk and control groups differed significantly on symptoms of depression, suicidality, anxiety and, as expected based on selection criteria—psychotic symptoms, with the at-risk group reporting significantly more symptoms. These variables were entered as covariates in the ANOVA to control for the effect of symptoms on cognition. When controlling for the influence of these symptoms on cognitive impairment, the results showed that the differences between groups were no longer significant for verbal learning when controlling for past depressive symptoms (*F* = 3.231, *p* = 0.075), present depressive symptoms (*F* = 2.347, *p* = 0.129), and symptoms of social phobia (*F* = 2.846, *p* = 0.095). However, the differences in composite score remained significant.

## Discussion

In this first population-based study of 15-year-olds with self-reported symptoms of psychosis, we found that the at-risk group performed worse than age- and gender- matched controls on a test battery of cognitive function. This accords with the majority of previous studies on individuals defined as CHR (13). This is the first time that this has been identified using a consensus test-battery in a young adolescent group who were not selected based on help-seeking behavior.

Another aim of the present study was to examine which specific cognitive domains are affected. Our results showed that the at-risk group performed significantly worse than controls on the composite score of the MCCB. However, the significant difference between groups in the verbal learning domain disappeared both when controlling for multiplicity and when controlling for both previous and present symptoms of depression and symptoms of social phobia. This underscores the importance of measuring both overall cognition and specific cognitive domains, as well as the importance of mapping psychiatric comorbidity. Results from previous studies on specific cognitive functions and impairment in older adolescents and young adults at risk for psychosis have been mixed. Some studies have found that individuals at-risk perform worse on processing speed and verbal learning and memory (15). Others find that declines on specific tests, such as Digit Symbol Coding that measures processing speed, predict transition to SSD (43). Yet others find impairment in attention, verbal learning and memory, working memory, verbal fluency and executive function, as well as a composite score in the at-risk group compared to healthy controls (17). There are, however, impairments in some specific domains that stand out in at-risk groups, such as verbal learning and memory (44, 45). Verbal learning and memory have been found to be more strongly associated with a genetic vulnerability to psychosis than other cognitive domains (46). Furthermore, verbal learning and memory impairments may not be associated with increase in symptomatic burden, but rather a trait that is related to vulnerability to developing psychosis and may be particularly predictive of future transition to psychosis (47).

It is reasonable to assume that our non-help-seeking participants experience less severe symptoms than CHR individuals who have sought help for their mental health problems. As such, the results suggest that worse performance on tests of verbal learning may be weakly related to psychotic symptoms. Nonetheless, psychotic experiences in early childhood predicts mental illness in middle childhood (48), and self-reported psychotic experiences in 11-year olds predicted SSD diagnoses at age 25 (49). Furthermore, our results expand upon previous findings and suggest that adolescents at-risk may be similar to children at FHR (14) in that they do not evidence impairments in verbal learning. It may be that impairments in verbal learning develop through early adolescence and become significant later in adolescence or young adulthood, in line with a neurodevelopmental model. Also, FHR for schizophrenia and a population-based general at-risk group may have different risk markers and etiologies. However, a neurodevelopmental model may be a good fit for both at-risk groups, with subtle differences and similarities in patterns of cognitive functioning in childhood and adolescence.

It is well established that individuals with SSD, both adolescents with early-onset schizophrenia and adults, perform below controls on tests of general intellectual abilities (50–52). The same has been found to be the case in CHR groups, who are selected based on help-seeking behavior, and FHR groups as well (45). Based on the results from our study, this may also be the case for younger adolescents at risk for psychosis who are not selected on the basis of help-seeking behavior. The degree of relative impairment in estimated general intelligence found in the at-risk group in the current study is less than found in adolescents with early-onset schizophrenia (51, 52). This may indicate that impairments worsen at or after onset of psychotic disorder. Another explanation is that not all in an at-risk group will develop SSD, and performance in at-risk groups may be both more heterogenous and less impaired than performance in established SSD groups.

Even though our data show a general cognitive deficit, overall, the at-risk group presented with mainly preserved cognitive functioning. The cognitive domains with the least differences between the at-risk and control group were working memory and reasoning. This is somewhat unexpected due to past research indicating that these domains are often among the most impaired in established schizophrenia populations (53), and also prevalent in CHR populations (54). However, some previous research supports the notion that working memory may be relatively spared early in the illness (55). It is possible that working memory and executive difficulties become apparent later in adolescence as frontal brain systems mature, or that impairments in these domains are more closely related to a worsening of psychotic symptoms (7).

## Strengths and limitations

The use of data from a large national cohort is a key strength of this study. There is a paucity of population cohort studies investigating at-risk groups and possible antecedents of psychosis. This is the first population cohort study where: (1) 15-year-olds have been identified as at-risk for psychosis through population survey items, (2) participants have been tested with a validated test battery that spans all core cognitive domains, and (3) an age- and gender matched control group was randomly selected from the same sample. A possible limitation in our study is the significant skew in gender, with 91% of the cohort being female, raising the question of generalizability to male adolescents at risk for psychosis. However, existing research find small differences between sexes in average performance on cognitive tests, both in general cohorts (56) and in at-risk groups populations (13, 14, 57). Based on the findings that girls generally outperform boys on verbal ability and memory tasks (58), one might expect significant impairments if our groups were more equally divided between sexes.

## Conclusion

Our results highlight a general cognitive deficit as central in early adolescence in an at-risk group with psychotic symptoms who are not selected based on help-seeking behavior. This implies that cognitive impairments can be identified early in life for at-risk individuals. Verbal learning may be valuable both as a risk-marker in itself and as a target of early remediation efforts. However, more research with larger samples are needed to investigate specific cognitive impairments and their relationship to other psychiatric symptoms in at-risk groups. In general, there is a need for long-term follow-up studies to investigate developmental trajectories and future SSD status, and how this relates to cognitive impairments in adolescence.

## Data availability statement

The datasets presented in this article are not readily available because the dataset is restricted by agreements with the Mother- Father and Child Study. Requests to access the datasets should be directed to MoBaadm@fhi.no.

## Ethics statement

The studies involving human participants were reviewed and approved by the Regional Committees for Medical and Health Research Ethics (ref. 2017/342). Written informed consent to participate in this study was provided by the participants or their legal guardian/next of kin.

## Author contributions

BR acquired funding and approval of the ethics committee. CM-H and CT carried out clinical testing. CM-H and CM performed the formal analyses. CM-H wrote the original draft. MØ, CM, FL, and BR contributed with supervision. All authors contributed to the conceptualization, study design, worked on, and approved the final manuscript.
